# Light to Moderate Alcohol Consumption Is Protective for Type 2 Diabetes Mellitus in Normal Weight and Overweight Individuals but Not the Obese

**DOI:** 10.1155/2014/634587

**Published:** 2014-07-22

**Authors:** Patricia A. Metcalf, Robert K. R. Scragg, Rod Jackson

**Affiliations:** ^1^Department of Statistics, University of Auckland, Private Bag 92019, Auckland 1142, New Zealand; ^2^Division of Epidemiology and Biostatistics, School of Population Health, University of Auckland, Private Bag 92019, Auckland 1142, New Zealand

## Abstract

*Objective*. To examine the association between alcohol consumption and risk of type 2 diabetes mellitus (T2DM) overall and by body mass index. *Methods*. Cross-sectional study of employed individuals. Daily alcohol intakes were calculated from a self-administered food frequency questionnaire by 5,512 Maori, Pacific Island, and European workers (3,992 men, 1520 women) aged 40 years and above. *Results*. There were 170 new cases of T2DM. Compared to the group with no alcohol consumption and adjusting for age, sex, and ethnicity, the group consuming alcohol had relative risks of T2DM of 0.23 (95% CI: 0.08, 0.65) in normal weight individuals, 0.38 (0.18, 0.81) in overweight individuals, and 0.99 (0.59, 1.67) in obese individuals. After further adjusting for total cholesterol, HDL-cholesterol, triglycerides, smoking habit, physical activity, socioeconomic status, body mass index, and hypertension, the relative risks of T2DM were 0.16 (0.05, 0.50) in normal weight individuals, 0.43 (0.19, 0.97) in overweight individuals, and 0.92 (0.52, 1.60) in overweight individuals. Across the categories of alcohol consumption, there was an approximate U-shaped relationship for new cases of T2DM. There was no significant association between alcohol consumption and IGT. *Conclusions*. Alcohol consumption was protective against diagnosis of T2DM in normal and overweight individuals but not in the obese.

## 1. Introduction

Some of the strongest predictors of type 2 diabetes mellitus (T2DM) are obesity and family history of diabetes [1, 2]. However, lifestyle factors are also important in the etiology of the disease, and several studies have shown that T2DM can be prevented through modifications to a healthier lifestyle, such as dietary modification [3–5] or increased physical activity [4–6].

Moderate alcohol consumption has been shown to be protective against cardiovascular disease. Recent meta-analyses have concluded that there is either a U-shaped [7, 8], J-shaped [9], or inverse association [10] between T2DM incidence and alcohol consumption. Furthermore, one prospective study reported that increased alcohol consumption over time was associated with lower risk of T2DM among initially rare and light drinkers [11].

Few studies have stratified the analyses of risk of T2DM and alcohol intake by body mass index (BMI). A U.S. study reported a U-shaped association between alcohol consumption and risk of T2DM in women with a body mass index (BMI) < 25 kg/m^2^ [4] but an inverse association in the overweight and the obese.

We have previously reported that individuals with newly diagnosed T2DM were inversely associated with income and were higher in Maori and Pacific adults compared to Europeans [12]. We assessed whether low to moderate alcohol consumption was associated with risk of T2DM both overall and separately in low-, middle- and high-BMI individuals in a cross-sectional study of middle-aged New Zealand men and women.

## 2. Methods

Between May, 1988, and April, 1990, 5,672 adult workers (4,103 men, 1569 women) were interviewed at 41 work sites in Auckland and five work sites in Tokoroa (response rate 67 percent). Detailed methods have been described previously [12]. Participants comprised 78.8% European, 7.7% Maori, 11.7% Pacific, and 1.8% Asian workers. Pacific participants were 53.7% Samoan, 10.7% Tongan, 6.8% Niuean, 26.5% from Cook Islands, and 2.3% from other Islands. Approval for the study was obtained from the Ethical Committee of the University of Auckland, and all participants gave informed consent. The median number of staff interviewed at each worksite was 72 (range 10–567). Management of 10 worksites refused giving permission for their staff to be interviewed (response rate for worksites was 82%).

All participants completed a self-administered questionnaire about sociodemographic factors, occupation, smoking habit, and current medication use. Ethnicity was self-defined. The New Zealand Socioeconomic Index (NZSEI) was calculated based on the occupation of the worker and spouse and a participant was assigned the higher of the two [13].

All participants were asked to fast from 10pm the evening before their interview. On the morning of the interview, participants were given a 75 g polycose (Abbott Laboratory) after the collection of fasting blood samples. A further blood sample was collected 2 hours after polycose load. Glucose tolerance status was evaluated by 2006 WHO criteria and 2011 ADA alternative criteria (using fasting glucose ≥ 7.0 mmol/L or 2 h post glucose load of ≥11.1 mmol/L for diabetes, fasting glucose < 7.0 mmol/L, and 2 h glucose between 7.8 and 11.0 mmol/L for IGT) [14, 15]. As all new cases of diabetes were diagnosed after 39 years of age, they were classified as T2DM rather than as type 1 diabetes mellitus.

All workers provided fasting blood specimens for glucose, cholesterol, HDL-cholesterol, and triglyceride estimations, and a blood sample two hours after a 75 g polycose load. Biochemical analyses were made using enzymatic methods. Interbatch coefficients of variation for normal control material, in percentages, were as follows: glucose, 3.9; cholesterol, 3.6; HDL-cholesterol, 3.4; and triglycerides, 4.7; and from abnormal control sera: glucose, 2.1; cholesterol, 3.6; and triglycerides, 5.4.

Leisure exercise was assessed using a three-month physical activity recall questionnaire that has been validated [16]. Vigorous activities were defined as those that made participant's breathe hard and moderate activities involved movement such as brisk walking. After a rest of 15 minutes or more, blood pressure was measured twice in the sitting position with a Hawksley random zero sphygmomanometer. Hypertension was defined as systolic blood pressure ≥ 140 mm Hg, and/or diastolic blood pressure ≥ 90 mm Hg, or current use of blood pressure lowering medications. Shoes and heavy clothes were removed for measurement of weight to the nearest 0.2 kg, and height to the nearest 0.5 cm. Body mass index was calculated as weight (kg) divided by the square of height (m).

Food intake over the previous 3 months was estimated by a 142-item food frequency questionnaire, which was filled in by participants at their home, and checked for errors and omissions at their interview the following morning. Natural serving sizes, such as the can of beer or serving of spirits, were used or published serve sizes [17]. The comprehensive version of the food composition tables [18] was used to calculate nutrient intakes. We have previously reported that alcohol intake from this food frequency questionnaire was valid (*r* = 0.84 in Europeans and *r* = 0.68 in Polynesians) and reproducible (*r* = 0.88 in Europeans and *r* = 0.83 in Polynesians) [19].

A generalised linear model was used to calculate adjusted means by body mass index and alcohol consumption groups. Relative risks and 95% confidence intervals were estimated using Poisson regression models, adjusted for potential confounders [20]. An overall model was fitted first to examine the interaction between alcohol intake and body mass index, and then separate models were fitted for three subgroups of participants defined as normal weight (BMI < 25 kg/m^2^), overweight (25 ≤ BMI < 30 kg/m^2^), and obese (BMI > 30 kg/m^2^). No alcohol consumption was taken as a reference group to estimate the relative risks for alcohol consumption in each BMI group. Alcohol consumption was also categorized into no alcohol consumption during the past three months, <5 g/day, <20 g/day and ≥20 g/day. All analyses were carried out using SAS Version 9.3 [21].

## 3. Results

A total of 5,672 participants were recruited into the study. Of these, 103 (1.8%) were excluded due to a previous history of diabetes mellitus, 53 (0.9%) who did not complete the food frequency questionnaire, and 4 (0.1%) with missing body mass index, leaving 5,512 (%). The median daily alcohol consumption was 4.8 g/day. The majority (75%) drank < 13 g/day of alcohol and 95% drank < 37 g/day.

Table 1 shows the characteristics of participants overall and by BMI levels. The mean age of participants was 48.8 years (range 40–78 years) and 87.6% consumed alcohol during the previous three months. Only 3.1% of individuals were diagnosed as new cases of T2DM and 4.5% had impaired glucose tolerance.

There were 34.4% individuals who were classified as normal weight, 43.7% as overweight, and 21.9% as obese (Table 1). Among normal weight individuals, the proportion of males was lower than overweight and obese individuals, as was the proportion of new cases of T2DM and impaired glucose tolerance. The group of obese individuals had the highest proportions of those with hypertension, current smokers, IGT, and new cases of T2DM. Mean fasting and 2-hour glucose levels, total cholesterol, triglycerides, and systolic and diastolic blood pressure increased with increasing BMI level (all *P* < 0.001). In contrast, HDL-cholesterol levels and NZSEI reduced with increasing BMI level (*P* < 0.001). Mean physical activity was significantly lower (*P* < 0.001) in the obese group compared to normal and overweight individuals.

Table 2 shows the characteristics of individuals by alcohol consumption group. There appeared to be a U-shaped relationship between alcohol consumption categories and new cases of T2DM, impaired glucose tolerance, and hypertension. Compared to the no alcohol consumption group, those consuming alcohol had lower 2-hour post-polycose glucose levels and NZSEI levels, and mean HDL-cholesterol and triglycerides levels increased over the alcohol consumption categories. Mean fasting glucose levels were lower in those who drank more than zero and less than 5 g/day, and geometric mean exercise levels were higher in those consuming 5 g/day or more. Participants consuming ≥20 g/day had higher mean total cholesterol and systolic and diastolic blood pressure levels. Lower mean body mass index levels were seen in those consuming 5 to less than 20 g/day.

Compared to the group with no alcohol consumption and after adjusting for age, sex, and ethnicity, the group consuming some alcohol during the past three months had the following relative risks of T2DM: normal weight individuals—0.22 (95% CI: 0.08, 0.64); overweight individuals—0.39 (0.18, 0.84); and 1.10 (0.65, 1.87) in obese individuals. After further adjusting for total cholesterol, HDL-cholesterol, triglycerides, smoking habit, physical activity, socioeconomic status, body mass index, and hypertension, the relative risks of T2DM were 0.16 (0.05, 0.50) in normal weight individuals, 0.43 (0.19, 0.97) in overweight individuals, and 0.92 (0.52, 1.60) in overweight individuals. The *P* value for the interaction term between BMI categories and alcohol consumption was <0.0001.

**Figure 1:**
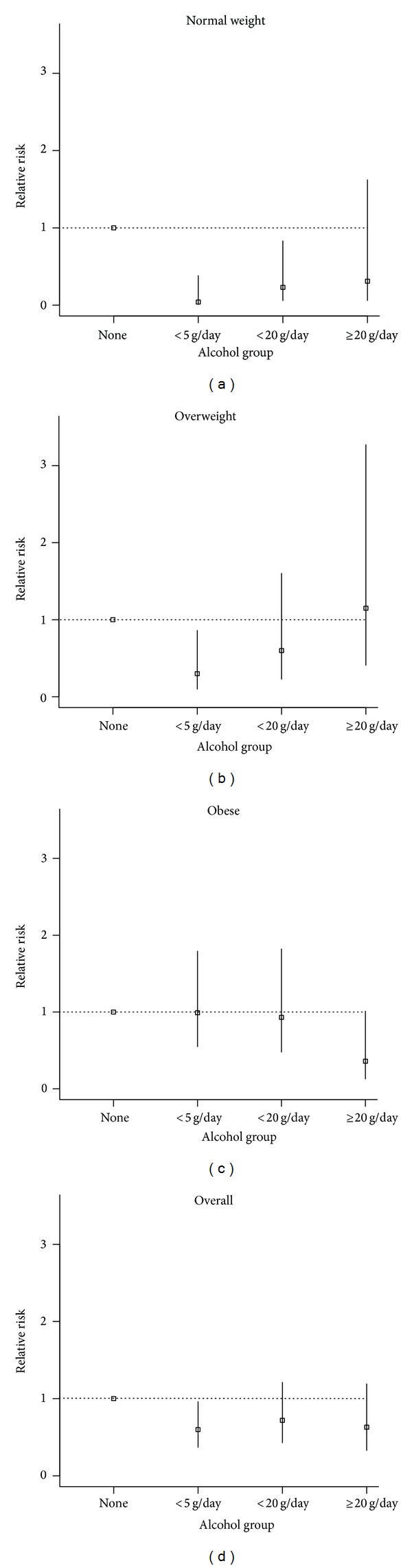
Relative risks and 95% confidence intervals for normal, overweight, and obese adults and overall by alcohol group.

The relative risks comparing no alcohol consumption to the various alcohol consumption groups are shown in Table 3 (column headed overall). After adjusting for age, gender, and ethnicity, the relative risk for T2DM overall was 0.53 (95% CI: 0.34, 0.82) in those drinking more than zero to <5 g/day, and 0.60 (0.37, 0.96) after also adjusting for current smoking habit, body mass index, total cholesterol, HDL-cholesterol, triglycerides, physical activity, socioeconomic status, and hypertension (referred to hereafter as all potential confounders).  Figure 1 shows the relative risks and 95% confidence intervals for normal, overweight, and obese adults and overall, by alcohol consumption group.

Relative risks associated with risk of T2DM by alcohol consumption and BMI groups are also shown in Table 3. There were lower relative risks for T2DM in those consuming more than zero to <5 g/day in both normal weight and overweight individuals, but not in obese individuals, after adjusting for age, gender, and ethnicity. After further adjusting for all potential confounders, similar relative risks were observed; however, the relative risk of type 2 diabetes in those consuming 5 to <20 g/day in normal weight individuals was also significantly lower (RR 0.23; 0.06, 0.83). In the overweight group, the relative risks for T2DM in the group consuming ≥20 g/day was 0.36 (0.13, 1.01), but this just failed to be statistically significant at the 5% level of significance.

There was no significant association between alcohol consumption and IGT after adjusting for all potential confounders with relative risks of 0.92 (0.37, 2.30) in normal weight individuals, 1.14 (0.56, 2.34) in overweight individuals, and 0.62 (0.38, 1.02) in obese individuals.

## 4. Discussion

In this study, low to moderate alcohol consumption over the past three months was associated with reduced risk of T2DM among normal weight and overweight individuals. However, there was no significant association between alcohol consumption and risk of T2DM in obese individuals. In the full model looking at no alcohol versus alcohol consumption, there was a highly statistically significant interaction between BMI group and alcohol intake. These associations persisted after adjustment for body mass index and other known and probable risk factors for T2DM.

However, in analyses that categorised alcohol into various groups, there appeared to be an approximate U-shaped relationship between alcohol consumption group and risk of T2DM in the full model and in the normal and overweight groups but not the obese group. It is possible that this is, in part, due to a lack of statistical power, or to the cut-off points chosen to classify alcohol intake.

Our findings of a possible U-shaped association between alcohol consumption and risk of T2DM in normal weight and overweight individuals have been described previously, but our finding of no association in the obese is not consistent with some other studies. A study of 84,941 female nurses reported a U-shaped association between alcohol consumption and risk of T2DM over 16 years of followup in those with a body mass index (BMI) < 25 kg/m^2^ [4] but an inverse association in the overweight and the obese. However, in the same study carried out 6 years earlier in 109,690 nurses aged 25 to 42 years they reported an inverse relationship between alcohol intake and incidence of T2DM in both the obese (BMI ≤ 30 kg/m^2^) and nonobese [22]. In contrast to the current study where diabetes status was determined from a glucose tolerance test, diabetes status was by self-report in initially employed women in these latter two studies [4, 22]. Moderate alcohol consumption was also associated with an inverse association with risk of self-reported T2DM in 16,154 European men and women which was stronger in the overweight and obese particularly at higher levels of alcohol intake [23]. However, the findings from a large study conducted over 12.1 years among US physicians [24] showed no such interaction. In that study there was an inverse association between alcohol consumption among individuals both above and below the median BMI of 24.4 kg/m^2^.

A Japanese study of 5,636 adults reported an increased risk of self-reported T2DM among low BMI individuals (BMI ≤ 22 kg/m^2^) and a decreased risk of T2DM among middle-BMI individuals (22.1–24.9 kg/m^2^) but no significant difference in high-BMI individuals (≥25 kg/m^2^) [25]. These findings in the overweight and obese are consistent with the current study; however, we could not assess the effect of BMI ≤ 22 kg/m^2^ due to the small number of individuals in this category. Another Japanese study of employed men [26] found that alcohol intake was associated with an increased risk of glucose tolerance test diagnosed T2DM in the lean but a protective effect in the overweight. In contrast, another Japanese study of 28,893 men reported a positive association between self-reported diabetes incidence over 10 years of followup and moderate to high alcohol (≥23 g/day) intake in men with BMI ≤ 22 kg/m^2^ but no significant association at >22 kg/m^2^ [27]. Alcohol consumption was also a positive risk factor for the development of fasting glucose diagnosed T2DM in overweight (BMI 23–<25 kg/m^2^) and obese (BMI 25–38.7 kg/m^2^) 2,500 young Korean males [28]. The prospective studies were carried out between 2 and 12 years.

Differences in drinking patterns or quality of the alcohol (e.g., drinking beer and wine versus drinking vodka and other spirits) may have contributed to these inconsistencies, in addition to incomplete adjustment for confounders or measurement error in alcohol assessment or the differences in the measurement of alcohol consumption levels (e.g., usual alcohol consumption versus recent alcohol).

In agreement with the findings in the current study, a study of over 8700 subjects from Micronesian and Mauritian populations, alcohol intake had no association with prevalence of impaired glucose tolerance (IGT) [29], nor was there a significant association in 6,362 Japanese men [26]. In contrast, a cross-sectional study of 3,128 Swedish men aged 35 to 56 years reported a reduced prevalence of IGT with moderate alcohol consumption levels [30], and another study reported that alcohol consumption was a positive risk factor for the development of IGT in overweight (BMI 23–<25 kg/m^2^) and obese (BMI 25–38.7 kg/m^2^) young Korean males [28]. Few other studies appear to have examined the association between IGT and alcohol consumption.

T2DM is caused by both insulin resistance and *β*-cell dysfunction [15, 31]. Several factors may explain the inverse or U-shaped association between moderate alcohol consumption and reduced risk of T2DM including increased insulin sensitivity with lower plasma insulin concentrations [32, 33], increased HDL-cholesterol [34] or due to the anti-inflammatory effect of alcohol [35, 36]. At higher alcohol consumption levels, body weight, blood pressure, and triglyceride concentrations increase [37–39]. Both moderate and acute alcohol consumption have also been to be reported to inhibit lipolysis with a reduction in free fatty acid levels [40, 41]. Such studies provide a possible physiological explanation for the inverse or U-shaped association between alcohol consumption and T2DM. It has also been hypothesized that regular moderate alcohol consumption promotes insulin sensitivity of skeletal muscle, resulting in a protective effect for risk of T2DM [42].

The possibility of differential reporting of alcohol consumption according to disease status was minimized as alcohol consumption data was collected prior to diagnosis of T2DM; therefore, drinking habits and recall of alcohol intake could not have been influenced by disease status. We relied on self-reported alcohol consumption data in this study, as did most other alcohol-related epidemiological studies, since it has been reported that other approaches are not practical in large studies [43]. Furthermore, the usefulness of simple self-administered questionnaires to reliably estimate alcohol consumption in these participants has been demonstrated [19] and was indirectly validated by the significant positive correlation with HDL-cholesterol. It is unlikely that misclassification accounted for the observed associations, since measurement error in observational studies tends to reduce or obscure a true association. A further limitation is that cross-sectional data cannot differentiate between cause and effect.

The key strength of the current study was that diabetes status was ascertained by oral glucose tolerance test rather than self-reported diabetes as has been used in the majority of previous studies.

In conclusion, results from the current study support the hypothesis that there is an inverse association between light to moderate alcohol consumption and the risk of T2DM in normal weight and overweight individuals but not in the obese. These results are limited to healthy working individuals consuming light to moderate amounts of alcohol and suggest that light to moderate alcohol consumption in addition to other known risk factors can modify the incidence of T2DM.

## Figures and Tables

**Table 1:** Characteristics of participants overall and by body mass index levels in employed men and women, 1988–1990.

	Overall	Normal	Overweight	Obese
*N*	5,512	1,896	2,410	1,206
Age (years)	48.8 (0.08)	48.5 (0.14)	49.2 (0.13)	48.5 (0.17)
Male (%)	72.4	64.9	80.3	68.5
No alcohol consumption	682 (12.4%)	199 (10.5%)	216 (9.0%)	267 (22.2%)
Alcohol (g/day)				
<5	2128 (38.6%)	780 (41.1%)	889 (36.9%)	459 (38.1%)
<20	1920 (34.8%)	685 (36.1%)	904 (37.5%)	331 (27.4%)
≥20	782 (14.2%)	232 (12.2%)	401 (16.6%)	149 (12.4%)
New type 2 Diabetes	170 (3.1%)	17 (0.9%)	44 (1.8%)	109 (9.0%)
IGT	248 (4.5%)	44 (2.3%)	104 (4.3%)	100 (8.3%)
Hypertension	997 (18.1%)	193 (10.1%)	435 (18.0%)	369 (30.6%)
Current cigarette smoker	1399 (25.4%)	474 (25.0%)	613 (25.4%)	312 (25.9%)
Ex-cigarette smoker	1845 (33.5%)	565 (29.8%)	871 (36.1%)	409 (33.9%)

Age, gender, and ethnicity adjusted				
BMI (kg/m^2^)	27.2 (0.06)	23.1 (0.05)	27.3 (0.04)∗∗∗	33.4 (0.07)∗∗∗
Fasting plasma glucose (mmol/L)	5.40 (0.01)	5.21 (0.05)	5.37 (0.02)∗∗∗	5.75 (0.03)∗∗∗
2 hour glucose (mmol/L)	4.93 (0.03)	4.59 (0.04)	4.85 (0.04)∗∗∗	5.62 (0.06)∗∗∗
Total cholesterol (mmol/L)	6.19 (0.02)	6.00 (0.03)	6.28 (0.02)∗∗∗	6.33 (0.04)∗∗∗
HDL-cholesterol (mmol/L)	1.26 (0.004)	1.35 (0.01)	1.24 (0.01)∗∗∗	1.15 (0.01)∗∗∗
Serum triglycerides (mmol/L)^1^	1.38 (1.01)	1.13 (1.02)	1.45 (1.02)∗∗∗	1.76 (1.03)∗∗∗
Systolic blood pressure (mm Hg)	123.6 (0.19)	119.3 (0.30)	123.9 (0.26)∗∗∗	129.8 (0.40)∗∗∗
Diastolic blood pressure (mm Hg)	76.6 (0.13)	73.6 (0.22)	76.8 (0.19)∗∗∗	80.9 (0.29)∗∗∗
Physical activity (min/day)^1^	16.2 (1.06)	17.6 (1.11)	17.2 (1.09)	12.7 (1.14)∗∗∗
NZSEI	46.2 (0.17)	47.4 (0.28)	46.1 (0.24)∗∗∗	44.6 (0.37)∗∗∗

***P < 0.001 versus normal weight individuals. Percentages are for that group (column).

^1^geometric mean (tolerance factor). IGT = impaired glucose tolerance. NZSEI = New Zealand socio-economic index.

**Table 2:** Characteristics of participants by alcohol consumption levels in employed men and women, 1988–1990.

	No alcohol	<5 g/day	<20 g/day	≥20 g/day
*N*	682	2,128	1,920	782
Age (years)	49.7 (0.24)	48.6 (0.13)∗∗∗	48.6 (0.14)∗∗∗	49.0 (0.22)∗
Male (%)		64.9	80.3	68.5
New diabetes	41 (6.0%)	49 (2.3%)	54 (2.8%)	26 (3.3%)
IGT	47 (6.9%)	87 (2.3%)	76 (4.0%)	38 (4.9%)
Hypertension	130 (19.1%)	346 (16.3%)	329 (17.1%)	192 (24.6%)
Current cigarette smoker	135 (19.8%)	521 (24.5%)	467 (24.3%)	276 (35.3%)
Ex-cigarette smoker	168 (24.6%)	629 (29.6%)	711 (37.0%)	337 (43.1%)

Age, gender, and ethnicity adjusted				
BMI (kg/m^2^)	27.8 (0.16)	27.1 (0.09)	27.0 (0.09)∗∗∗	27.4 (0.15)
Fasting plasma glucose (mmol/L)	5.44 (0.04)	5.35 (0.02)∗	5.40 (0.02)	5.48 (0.03)
2-hour glucose (mmol/L)	5.23 (0.09)	4.89 (0.04)∗∗∗	4.85 (0.04)∗∗∗	4.97 (0.07)∗∗
Total cholesterol (mmol/L)	6.12 (0.05)	6.16 (0.03)	6.16 (0.03)	6.43 (0.04)∗∗∗
HDL-cholesterol (mmol/L)	1.16 (0.01)	1.20 (0.01)∗∗∗	1.30 (0.01)∗∗∗	1.39 (0.01)∗∗∗
Serum triglycerides (mmol/L)^1^	1.32 (1.04)	1.35 (1.02)	1.39 (1.02)∗	1.56 (1.04)∗∗∗
Systolic blood pressure (mm Hg)	123.6 (0.53)	122.4 (0.29)	124.0 (0.30)	125.9 (0.48)∗∗
Diastolic blood pressure (mm Hg)	75.9 (0.38)	76.3 (0.21)	76.8 (0.22)	77.8 (0.34)∗∗
Physical activity (min/day)^1^	12.7 (1.19)	14.9 (1.10)	18.7 (1.10)∗∗∗	18.1 (1.17)∗∗
NZSEI	44.2 (0.46)	46.5 (0.25)∗∗∗	46.8 (0.27)∗∗∗	45.8 (0.42)∗∗∗

*0.01 < *P* < 0.05; **0.001 < *P* < 0.1; ***P ≤ 0.001 compared to no alcohol group. Percentages are for that column.

^1^Geometric mean (tolerance factor). IGT: impaired glucose tolerance; NZSEI: New Zealand socioeconomic index.

**Table 3:** Multivariate relative risks (95% confidence interval) for new cases of type 2 diabetes mellitus in employed men and women, 1988–1990.

	Overall	Normal	Overweight	Obese
Alcohol consumption				
None^1^	1.0 (Referent)	1.0	1.0	1.0
<5 g/day	0.53 (0.34, 0.82)	0.05 (0.01, 0.39)	0.20 (0.07, 0.54)	1.00 (0.58, 1.74)
<20 g/day	0.69 (0.43, 1.08)	0.32 (0.10, 1.04)	0.43 (0.18, 1.03)	1.08 (0.59, 1.98)
≥20 g/day	0.80 (0.46, 1.37)	0.64 (0.17, 2.42)	0.74 (0.30, 1.84)	0.76 (0.33, 3.72)
None^2^	1.0 (Referent)	1.0	1.0	1.0
<5 g/day	0.60 (0.37, 0.96)	0.04 (0.01, 0.38)	0.30 (0.10, 0.86)	0.99 (0.55, 1.79)
<20 g/day	0.72 (0.43, 1.21)	0.23 (0.06, 0.83)	0.60 (0.23, 1.60)	0.93 (0.48, 1.82)
≥20 g/day	0.63 (0.33, 1.19)	0.31 (0.06, 1.62)	1.15 (0.41, 3.27)	0.36 (0.13, 1.01)

^1^Adjusted for age, sex, and ethnicity.

^2^Adjusted for age, sex, ethnicity, smoking habit, body mass index, total cholesterol, HDL cholesterol, hypertension, physical activity, and socioeconomic status.
